# Supplementary material for: Molecular mechanisms and hotspots of pH sensing in ASIC1a revealed by computational and functional analysis
Source: Commun Biol. 2025 Nov 26;8:1692. doi: 10.1038/s42003-025-09090-9 (PMC12658276; doi:10.1038/s42003-025-09090-9)
Supplement: Supplementary file 2 — Supplementary information [file 42003_2025_9090_MOESM2_ESM.pdf]

Supporting information

Molecular mechanisms and hotspots of pH sensing in ASIC1a revealed by computational and functional analysis

Olivier Bignucolo[1,2,4], Ophélie Molton[3,4], Ivan Gautschi[3] and Stephan Kellenberger[3]

[1]Swiss Institute of Bioinformatics, Basel, Switzerland.

[2]miri dynamics, Basel, Switzerland.

[3]Department of Biomedical Sciences, University of Lausanne, Lausanne, Switzerland.

[4]These authors contributed equally: Olivier Bignucolo, Ophélie Molton.

Supplementary Methods

Alleviating the counterion effect when using the PBEQ solver

Since proteins are highly dynamics biomolecules, we designed a strategy based on the calculation of the pKas using structures extracted from short MD simulations, as described in details in the method sections and in previous publications [1,2].

It is expected that during an MD simulation, counterions will approach and interact with charged or hydrophilic protein atoms, and this will affect the pKa calculation. The basic idea is simple: an ion displaces a charged entity located within its reach. The PBEQ solver, which treats the solvent as a homogenous medium with a given dielectric constant (ε), calculates the charge spatial distribution(ρ) around this entity (see section Derivation of the Poisson-Boltzmann Equation). Since the solver "ignores" that the displacement is due to a charged local ion, the result will be biased. If, for example, a Lys amino group is pushed by a sodium ion towards a negatively charged side chain, the calculated ρ will be more negative than the 'true' (ρ), which incorporates a strong close positive charge. We estimated the amplitude of this perturbation on the pKa calculation and present here the data that led us to incorporate in the structure analyzed by the PBEQ solver monovalent counterions located within 3 Å of the protein in the selected frame.

Supplementary Table 4 reports the identity of the selected acidic residues, all located at the surface of hASIC1a and the distance r between their carboxylic group and the closest sodium ion. It reports the calculated pKas when the solver 'knew' and when it 'ignored' the counterion, and finally the difference, or bias, between these two values and the **supplementary Fig. 9** shows the fit of the Coulomb law on these points.

It follows a few conclusions, remarks, and limitations.

1) When the distance becomes larger than 3 Å, the bias becomes smaller as 0.5 pKa units, thus negligible since the solver accuracy lies within this range.

2) The fit to the Coulomb law, implying a simplification of the system in a two-point charge one, may not be the perfect choice since the constant C should be zero. However, as reported in point 1, our goal was solely to identify rapidly a reasonable offset.

3) Adding explicit ions to the system, whereas the solver is instructed of a precise ion concentration (150 mM) used to estimate ε will results in an overestimation of the ion concentration. In most frames the number of ions located within 3Å of the protein was between 0 and 10. We hope that this number was sufficiently small to not affect too much the ε estimation.

4) In this study we did not conduct the same analysis for divalent ions. For Divalents, the threshold was doubled, thus we considered divalent ions within 6 Å. It was however not tested, whether this threshold is sufficient.

5) Although not explicitly described, this counterion incorporation was already performed in our previous paper involving pKa calculations of proteins [1,2].

Supplementary Table 1. Available information on titratable residues not functionally analyzed in this study

Residue	Effect	Mut	ΔpH50	Structural interpretation of not tested residues
Lys105	0	C-AF488[de]	0.13, 0.12	
Asp107	Eff.	Q[a]	-0.88	
Glu113	0	Q[c] ;C-AF488[d]	-0.025; 0.23	
Glu123	Eff.	Q[a]	0.30	
Asp126				Finger loop, exposed
Asp131				Finger loop, exposed
Glu132				α2 helix, exposed
Lys133	0	C-AF488[de]	0.25	
Glu136	Eff.	Q[c]; C-AF488[d]	-0.008, 0.33	
Asp140				α2 helix, exposed
Lys141				α2 helix, exposed
Lys148				α2 helix-α3 helix loop exposed
Lys150				α2 helix-α3 helix loop exposed
Glu156				α3 helix, exposed
Asp159				α3 helix, parallel to K193
His163	0	N[a], C[f]	0.01, -0.15	
Asp164	0	N[c]	-0.11	
Asp167	0	N[a]	0.06	
Glu177	0	Q[c]	0.05	
Glu182	Eff.	Q[a]	0.18	
Asp183	0	N[c]	-0.10	
Lys185				β ball, exposed, towards D202
Asp202	Eff.	N[a]	0.18	
Lys208				palm β5-β6 loop, exposed towards lower palm
Asp223	0	N[a], N[c]	0, 0.02	
Asp227	0	N[a]	-0.07	
Glu228	0	Q[a], Q[c]; C,[d]	0.04, -0.13, -0.23	
Glu235	Eff.	Q[a], Q**[c], C-AF488[de]	-0.22, 0.003, 0.16, 0.20	
Asp237	Eff.	N[a], A[i], K[g]	-0.44, 0.05, 0.57	
His250	0	N[a]	0.04	
Asp253	Eff.	N[a]	0.26	
Asp296				thumb α4-α5 loop, exposed, other charged residues close
Asp300				thumb α4-α5 loop, exposed, other charged residues close (D313, R317)
Asp303	Eff.	N[a], N[c]	0.27, -0.10	
Asp313	0	N[c]	0.05	
Glu321	0	Q[c]	0.01	
Asp333				α4-α5 loop, exposed
Glu340				α5, end
Glu344	0	Q[a], Q[c]	-0.02, -0.04	
Lys356	Eff.	M[a], AzF[h]	-0.01, -0.53	
Glu359				thumb-palm loop, exposed
Lys380	Eff.	M[a]	0.30	
Lys392	Eff.	M[a], C-AF488[de]	-0.20, 0.17, 0.31	
Lys393	0	M[a]	0.01	
Lys396	Eff.	M[a]	-0.49	
Glu398	0	Q[a]	0.03	
Glu403	Eff.	Q[a]	0.17	
Glu421	0	C**(2)R	0.01	
Lys423	Eff.	M[a]	-0.60	
Lys424	0	C-AF488[e]	0.23	
Glu427	Eff.	G[b], C-AF488[d]	-0.17, 0.40	

ΔpH50 = pH50(Mutant)-pH50(WT); **, mutation to several residues tested; ND, not determined; C-AF488, Cys mutation, modified with fluorophore AF488; AzF, crosslinker [3]; LOF, loss of function (in ASIC2a); the upper case single letters indicate residues (single letter code) by which the WT residue was replaced. In "Other studies, "Eff." indicates that the pH50 shift was ≥ 0.15 of a conservative mutation, or ≥0.3 of a non-conservative mutation in ASIC1a. References: a, [4]; b, [5]; c, [6]; d, [7]; e, [8]; f, [9]; g, [10]; h, [3]; i, [11]

Supplementary Table 2. pH50 and pHD50 values

Location	Residue	Mutation	pH50	N	pHD50	N
Palm	WT		6.51 ± 0.01	137	7.15 ± 0.01	110
	H70	H70N	6.61 ± 0.02	8	7.20 ± 0.01	9
	H72	H72N	6.50 ± 0.03	10	7.12 ± 0.02	5
	H73	H73Q	6.30 ± 0.02	8	7.14 ± 0.01	8
		H73A	6.21 ± 0.03	8	7.04 ± 0.01	8
		H73V	6.29 ± 0.03	7	7.00 ± 0.01	8
		H73F	6.10 ± 0.03	7	6.95 ± 0.02	21
		H73D	5.99 ± 0.10	9	7.16 ± 0.02	7
		H73E	5.81 ± 0.06	14	7.09 ± 0.02	20
		H73K	6.40 ± 0.02	9	7.02 ± 0.02	12
	K76	K76Q	6.50 ± 0.04	7	7.24 ± 0.01	8
	D78	D78N	6.17 ± 0.04	14	7.07 ± 0.01	8
		D78Q	6.15 ± 0.03	8	7.17 ± 0.01	7
		D78M	6.09 ± 0.02	7	7.21 ± 0.01	8
		D78V	6.02 ± 0.06	11	7.15 ± 0.01	7
		D78E	6.62 ± 0.02	6	7.16 ± 0.02	11
		D78R	6.19 ± 0.03	8	7.02 ± 0.04	17
		D78H	6.22 ± 0.02	7	7.13 ± 0.01	7
	E79	E79A	6.07 ± 0.11	7	7.34 ± 0.01	9
	H173	H173N	6.53 ± 0.03	10	7.23 ± 0.02	6
		H173A	6.47 ± 0.04	9	7.28 ± 0.01	9
		H173E	6.67 ± 0.06	7	7.26 ± 0.01	9
		H173K	6.66 ± 0.02	9	7.31 ± 0.01	8
	E277	E277Q	6.15 ± 0.04	10	7.15 ± 0.01	7
		E277A	6.12 ± 0.02	10	7.21 ± 0.01	7
		E277D	6.45 ± 0.05	9	7.22 ± 0.01	7
	K374	K374Q	6.24 ± 0.03	16	6.99 ± 0.02	15
		K374A	6.14 ± 0.06	8	7.01 ± 0.03	6
		K374M	6.40 ± 0.05	15	7.13 ± 0.01	8
		K374V	6.15 ± 0.04	7	6.83 ± 0.02	23
		K374E	6.32 ± 0.05	17	6.89 ± 0.02	10
		K374R	6.58 ± 0.01	8	7.05 ± 0.02	9
		K374H	6.32 ± 0.04	11	6.90 ± 0.01	8
	E375	E375Q	6.30 ± 0.05	12	6.93 ± 0.01	11
		E375N	6.20 ± 0.04	10	6.83 ± 0.04	8
		E375A	6.38 ± 0.02	12	6.84 ± 0.02	6
		E375D	6.45 ± 0.02	7	7.02 ± 0.03	7
		E375R	5.83 ± 0.05	12	6.77 ± 0.02	22
		E375K	5.67 ± 0.02	8	6.72 ± 0.02	8
	E413	E413Q	6.11 ± 0.05	15	7.03 ± 0.01	8
		E413N	6.20 ± 0.05	16	6.89 ± 0.02	8

		E413A	6.20 ± 0.02	11	6.77 ± 0.03	5
		E413D	6.40 ± 0.03	10	7.20 ± 0.01	6
		E413R	5.96 ± 0.04	10	6.76 ± 0.02	17
		E413K	6.08 ± 0.04	8	6.84 ± 0.02	10
	E418	E418Q	6.02 ± 0.05	7	7.04 ± 0.01	9
		E418N	5.89 ± 0.10	8	7.00 ± 0.02	28
		E418A	5.67 ± 0.09	7	7.10 ± 0.02	6
		E418D	6.36 ± 0.08	10	6.83 ± 0.02	16
		E418R	6.19 ± 0.02	8	7.11 ± 0.02	29
Acidic pocket	K211	K211Q	6.02 ± 0.04	11	7.14 ± 0.01	8
		K211A	6.28 ± 0.03	8	7.20 ± 0.00	7
		K211M	6.18 ± 0.05	13	7.24 ± 0.01	7
		K211E	6.03 ± 0.04	9	7.05 ± 0.01	8
		K211R	6.05 ± 0.05	10	7.13 ± 0.01	7
		K211H	6.01 ± 0.06	13	7.26 ± 0.01	8
	E219	E219Q	6.36 ± 0.01	11	7.06 ± 0.01	8
		E219A	6.17 ± 0.02	8	6.91 ± 0.05	7
		E219D	6.22 ± 0.05	16	7.04 ± 0.02	8
		E219K	6.19 ± 0.02	9	6.88 ± 0.02	9
		E219H	6.45 ± 0.03	15	7.08 ± 0.02	13
	E238	E238Q	6.26 ± 0.05	12	7.07 ± 0.02	13
	E242	E242Q	6.33 ± 0.03	12	7.04 ± 0.01	8
		E242A	6.22 ± 0.01	9	6.95 ± 0.02	7
		E242D	6.18 ± 0.05	11	7.08 ± 0.01	6
		E242K	6.16 ± 0.04	11	6.98 ± 0.01	8
	K246	K246Q	6.09 ± 0.05	7	6.88 ± 0.01	9
		K246A	6.34 ± 0.07	7	7.09 ± 0.04	7
		K246M	6.32 ± 0.01	7	7.01 ± 0.01	8
		K246V	6.20 ± 0.05	12	7.03 ± 0.01	7
		K246E	6.13 ± 0.02	8	6.89 ± 0.04	12
		K246R	6.56 ± 0.01	9	7.16 ± 0.01	7
		K246H	6.08 ± 0.03	13	6.85 ± 0.02	11
	D259	D259N	6.53 ± 0.01	8	7.06 ± 0.02	9
	K343	K343Q	6.38 ± 0.06	7	7.15 ± 0.01	5
	D347	D347N	6.26 ± 0.05	8	6.99 ± 0.02	26
		D347E	5.94 ± 0.06	13	6.81 ± 0.02	25
		D347K	5.44 ± 0.09	12	6.88 ± 0.02	27
	D351	D351N	6.70 ± 0.01	7	7.28 ± 0.01	8
		D351E	6.29 ± 0.02	7	6.97 ± 0.02	11
		D351K	6.13 ± 0.07	11	7.05 ± 0.02	11
	E355	E355Q	6.47 ± 0.05	8	7.17 ± 0.02	21
	D409	D409N	6.60 ± 0.03	7	7.40 ± 0.04	25
		D409A	6.41 ± 0.03	14	7.08 ± 0.10	6
		D409K	6.30 ± 0.04	12	7.24 ± 0.02	8
	H110	H110N	6.48 ± 0.03	10	7.14 ± 0.02	11

		H110K	6.11 ± 0.08	12	7.04 ± 0.02	12
	K291	K291Q	6.50 ± 0.03	15	7.17 ± 0.01	7
	D298	D298N	6.56 ± 0.04	12	7.11 ± 0.01	9
	E315	E315Q	6.59 ± 0.05	8	7.36 ± 0.01	8
		E315K	5.23 ± 0.06	9	6.93 ± 0.02	9
		E315H	6.50 ± 0.04	8	7.16 ± 0.01	8
	H329	H329N	6.32 ± 0.02	10	7.01 ± 0.02	9
Finger and thumb		H329A	6.30 ± 0.02	7	6.98 ± 0.02	7
		H329M	6.39 ± 0.03	8	7.12 ± 0.01	6
		H329V	6.03 ± 0.03	8	6.85 ± 0.01	6
		H329F	6.26 ± 0.07	8	7.09 ± 0.01	8
		H329E	6.17 ± 0.03	8	6.95 ± 0.02	17
		H329K	5.82 ± 0.03	8	6.91 ± 0.01	10
	D357	D357N	5.96 ± 0.02	8	6.91 ± 0.01	10
		D357A	5.80 ± 0.04	7	6.88 ± 0.01	14
		D357K	5.48 ± 0.05	11	6.89 ± 0.02	8
	E364	E364Q	6.56 ± 0.01	8	7.13 ± 0.02	9
β-ball	E97	E97Q	6.40 ± 0.05	24	7.31 ± 0.01	8
		E97K	6.26 ± 0.06	11	7.17 ± 0.02	7
	K193	K193Q	6.47 ± 0.05	6	7.14 ± 0.01	16
	E254	E254Q	6.46 ± 0.03	7	7.03 ± 0.01	9
Knuckle	K384	K384Q	6.53 ± 0.02	9	7.09 ± 0.01	8
		K384M	6.40 ± 0.06	10	7.12 ± 0.02	6
		K384R	6.54 ± 0.01	7	7.11 ± 0.01	8
		K384H	6.60 ± 0.04	6	7.09 ± 0.03	11
	K388	K388Q	6.50 ± 0.01	8	7.05 ± 0.01	7
		K388M	6.58 ± 0.01	8	7.11 ± 0.02	8
		K388R	6.63 ± 0.02	6	7.12 ± 0.01	7
		K388H	6.52 ± 0.05	7	7.08 ± 0.01	7
Transmembrane	E63	E63Q	6.53 ± 0.09	7	7.19 ± 0.01	8
	D434	D434N	6.40 ± 0.07	8	7.20 ± 0.01	7
	D455	D455N	6.47 ± 0.09	7	7.22 ± 0.02	7

Double mutation	pH50	N	pHD50	N
H73D/D78H	6.24 ± 0.03	7	7.27 ± 0.01	5
K173E/E219K	6.58 ± 0.03	8	7.26 ± 0.02	8
K374E/E375K	6.34 ± 0.03	8	6.82 ± 0.04	17
K374E/E413K	6.38 ± 0.01	7	6.75 ± 0.01	6
K211D/D315K	5.13 ± 0.07	7	7.02 ± 0.01	5
K211D/D351K	5.97 ± 0.09	8	7.14 ± 0.02	13
K246E/E242K	5.93 ± 0.02	7	6.73 ± 0.01	7
K246D/D409K	6.09 ± 0.05	14	7.22 ± 0.01	8
H329E/E315K	6.24 ± 0.02	8	7.09 ± 0.01	8

Data are presented as mean ± SEM, with indication of n.

Supplementary Table 3. Distances between residues

	Distance in Å			Functional evidence for interaction in	
Pair	Closed	Open	Desensitized	Activation	SSD
H73-D78	4.15	5.54	3.13	Yes (double like D78H)	Yes (not additive)
H173-E219	5.76	5.41	5.91	No (additive)	? (interaction or H173E dominant)
K374-E375	2.58	3.04	3.73	Yes	Yes
K374-E413	5.21	5.81	7.00	Yes	No (additive)
K211-E315	5.11	6.37	5.49	No (no rescue)	? (partial rescue)
K211-D351	5.52	11.27	11.25	No (no rescue)	? (partial rescue)
K246-E242	6.85	10.07	10.00	No (additive)	No (additive)
K246-D409	6.79	7.26	6.89	No (no rescue)	? (partial rescue or D409K dominant)
H329-E315	4.58	5.47	5.77	Yes (rescue)	Yes (rescue)

Distances were measured between the closest side chain heavy atoms in the structural models of the three ASIC conformations. The average of the 3 distances is indicated here. ?, it is not clear whether there is an interaction.

Supplementary Table 4. Effect of Na^+ proximity on pKa

Residue number	Residue name	Distance to ion (Å)	pKa ion included	pKa ion omitted	ΔpKa
242	glu	11.20	7.17	7.33	-0.16
242	glu	9.20	7.27	7.38	-0.11
355	glu	9.00	6.52	6.60	-0.08
355	glu	7.80	6.58	6.63	-0.05
235	glu	7.00	3.15	3.32	-0.17
235	glu	5.00	3.05	3.24	-0.19
237	asp	3.15	3.46	5.46	-2.00
344	glu	3.00	3.18	4.57	-1.39
219	glu	2.60	4.54	6.81	-2.27
219	glu	2.50	4.57	6.58	-2.01
237	asp	2.50	3.69	5.37	-1.68
344	glu	2.45	3.32	4.60	-1.28

See explanation in Supporting Methods.

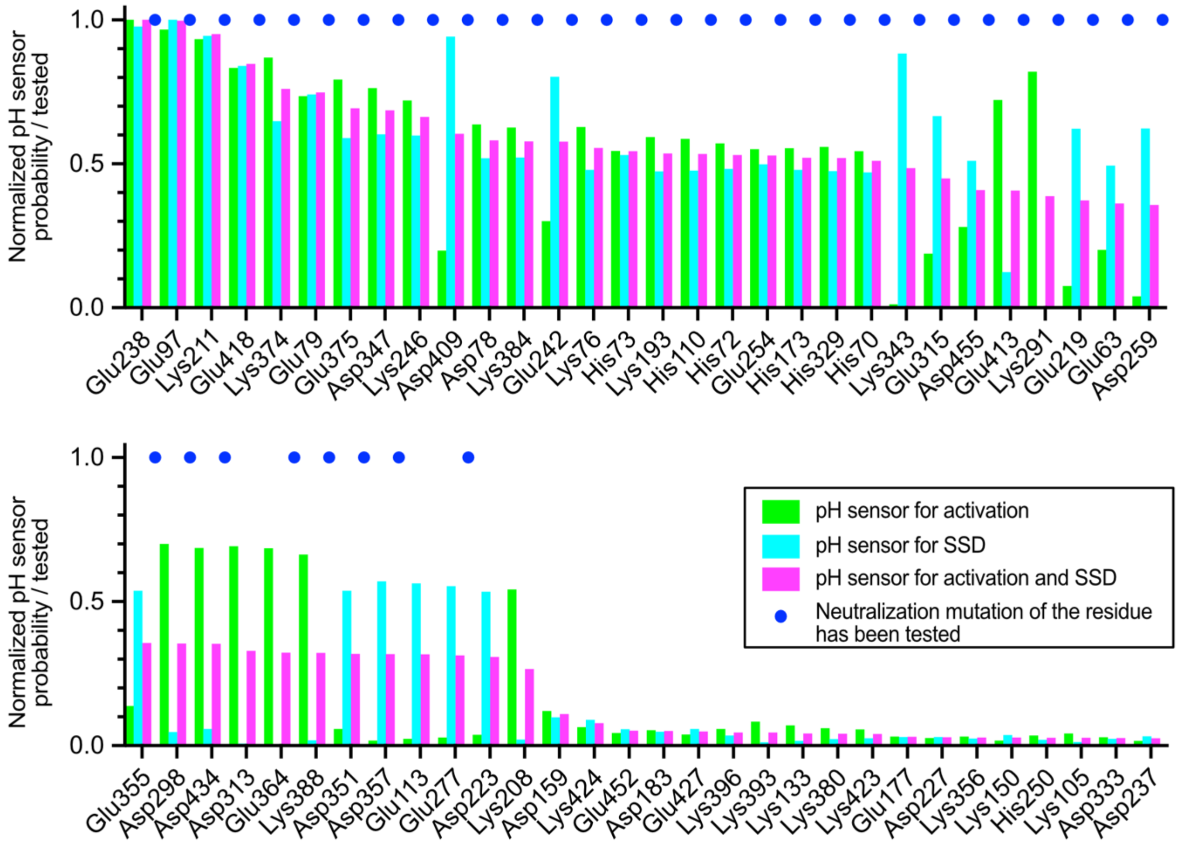

Supplementary Fig. 1 Ranking of the probability of titratable residues as pH sensors. The probability of being a pH sensor was calculated based on the determined pKa values, as described in the Results and *Methods*. Normalized data for the probability of being a pH sensor for activation (green), for SSD (cyan) and for both activation and SSD (pink) are plotted as bars, ranked from the highest to the lowest values, showing the 60 best-ranked residues. The blue circles at level 1 indicate that the pH dependence of a conservative mutation to a non-titratable amino acid of the given residue has been generated and functionally assessed. Some intermediately high-ranking residues were not functionally tested because previous studies had shown that their mutation did not affect the pH dependence.

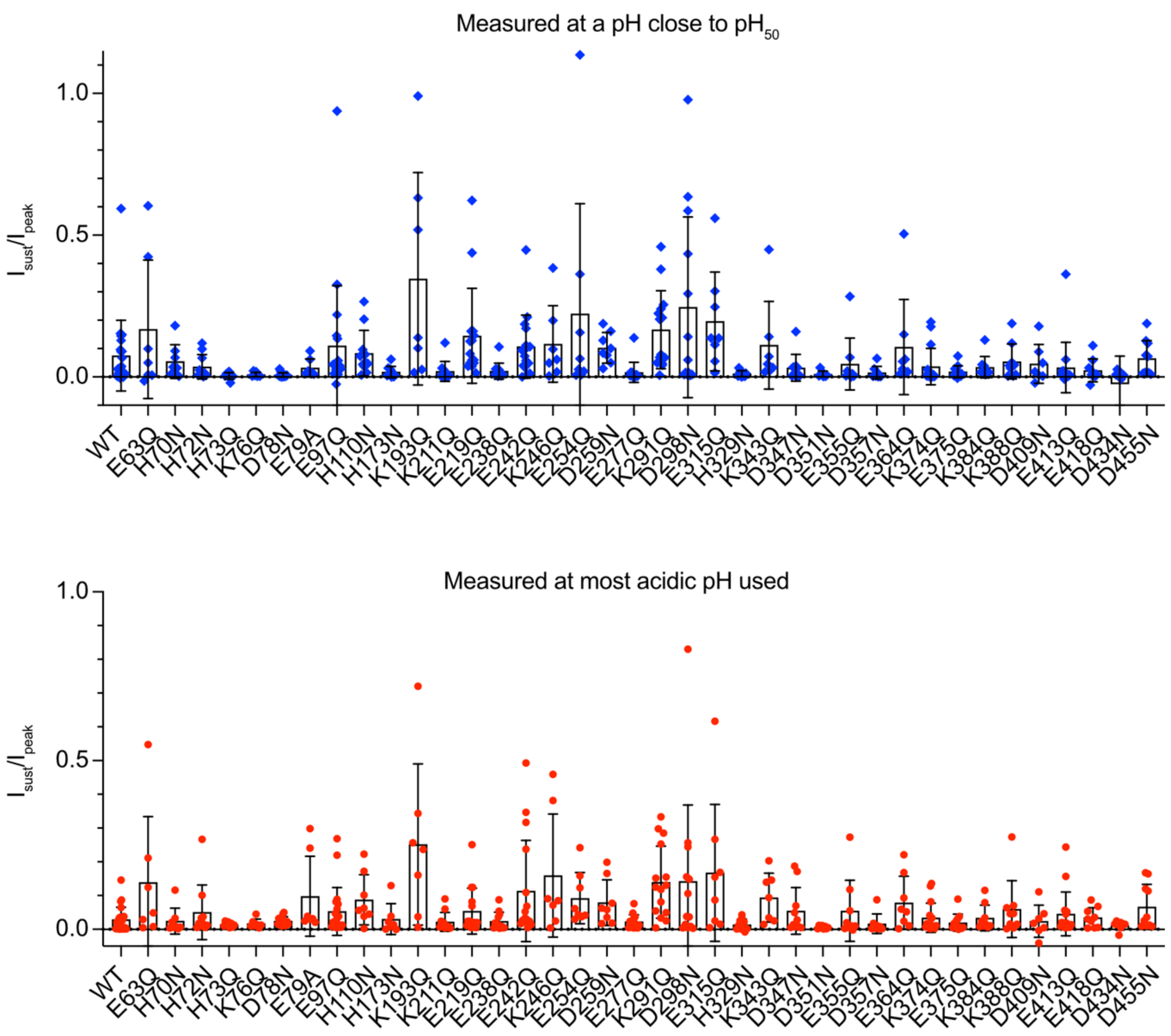

Supplementary Fig. 2 Sustained current fraction of neutralization mutations. The sustained current/peak current ratio (Isust/Ipeak) was measured at a pH close to the pH50 of the channel (upper panel) and at the most acidic pH, generally 4.5 or 5 (bottom panel); n=7-23.

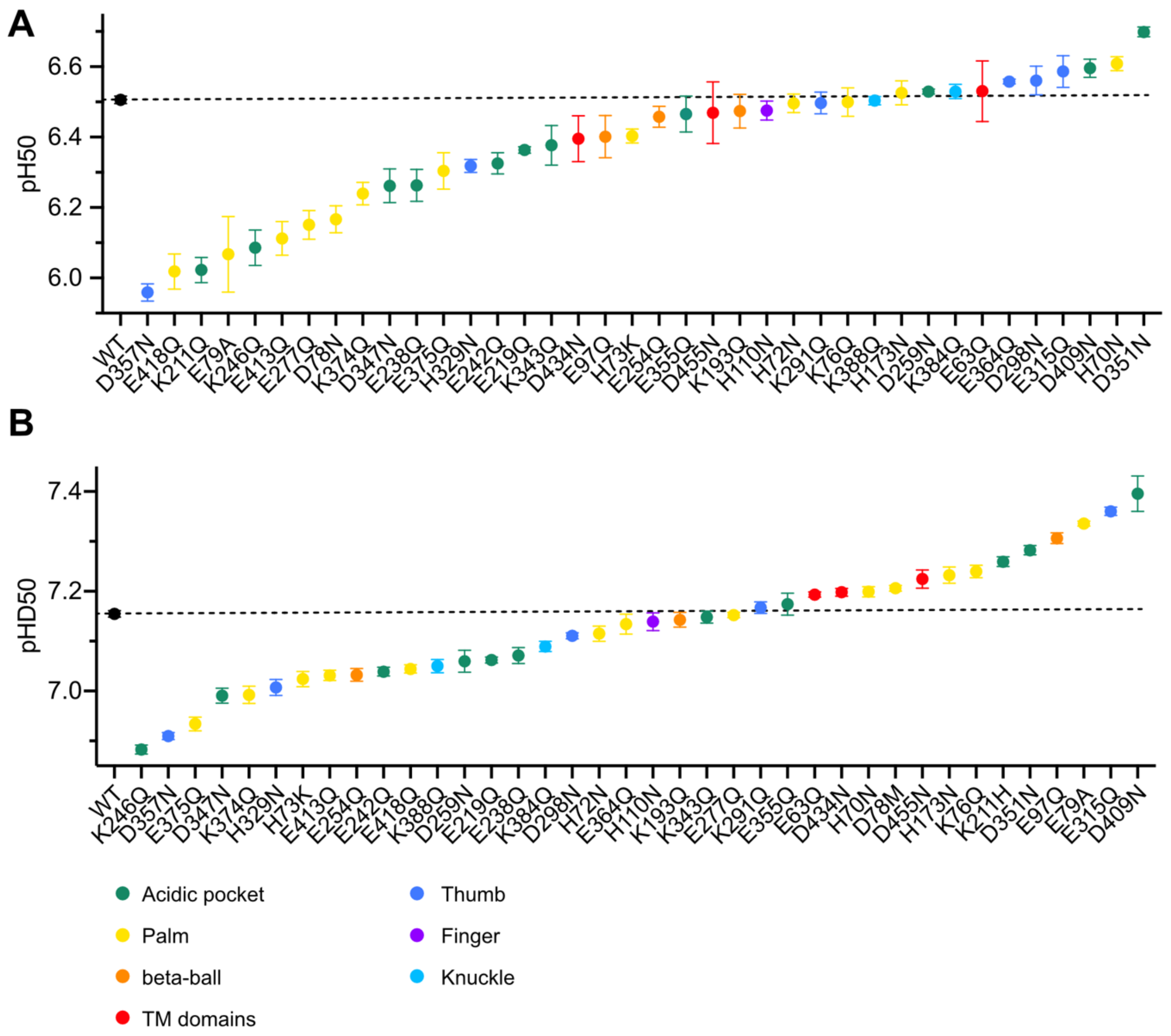

Supplementary Fig. 3. Ranking of pH_{50} and pHD_{50} shifts of conservative mutations to non-titratable residues. The pH_{50} (**A**) and pHD_{50} values (**B**) are ranked based on their value, comparing them to the corresponding WT value shown as black symbol and the vertical dashed line. The symbols representing the mutants are colored according to their domain, using the indicated color code. This color code is green for the AcP and the same color as that used in the structural images in other domains.

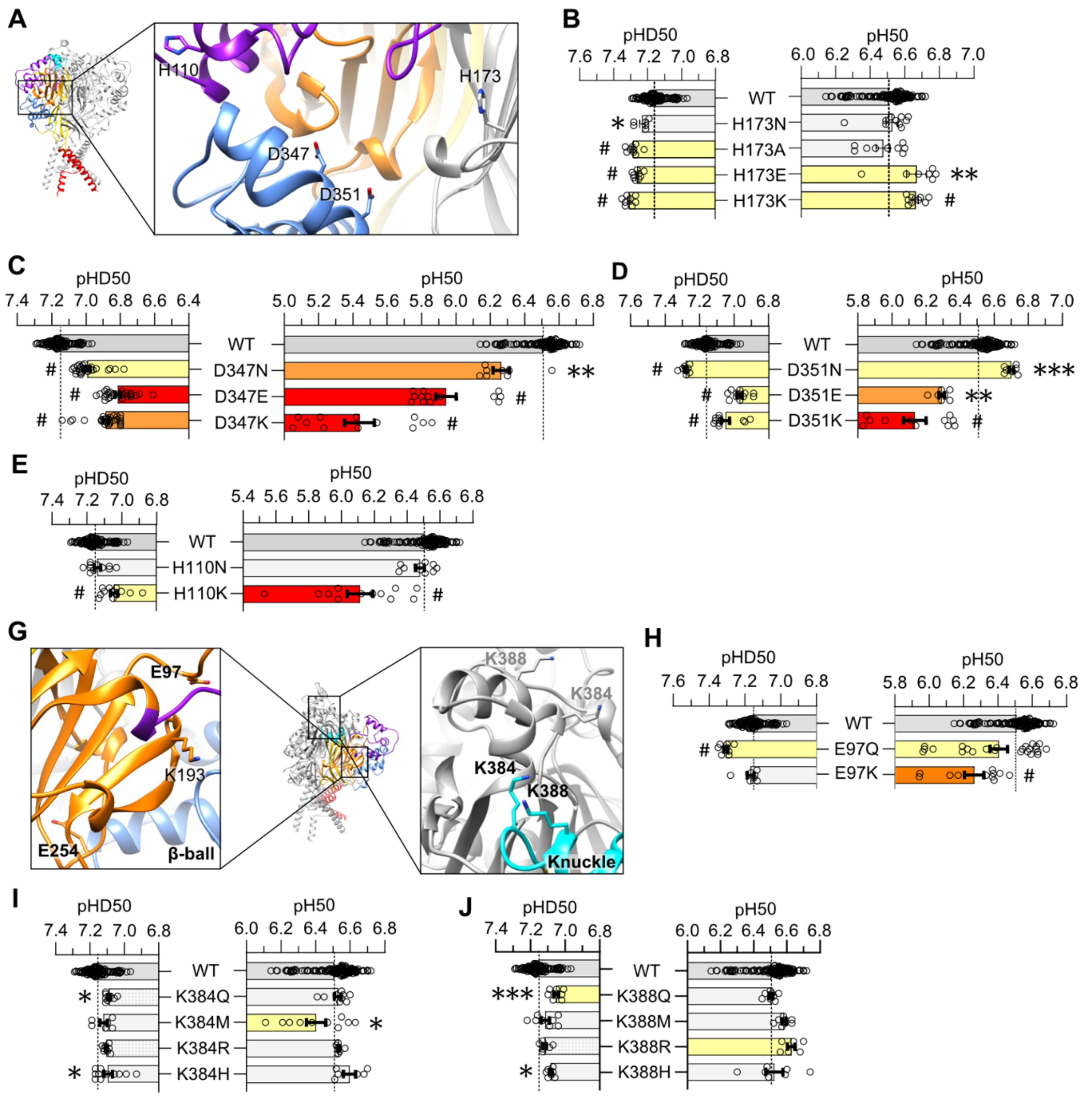

Supplementary Fig. 4. Functional investigation of putative pH sensors of several ASIC1a domains. (**A**) Structural image showing the location of H110, H173, D347 and D351, based on the structural model of closed ASIC1a (5WKU). **(B-E)**, pH_{50} and pHD_{50} values of WT ASIC1a and mutants of residue H173 (**B**), D347 (**C**), D351 (**D**) and H110 (**E**). (**G**) Structural image of the transmembrane segments in the closed conformation (based on 5WKU), showing the location of the predicted pH sensors, with the putative pH sensors labeled in bold. (**H-J**), pH_{50} and pHD_{50} values of WT ASIC1a and mutants of residue E97 **(H)**, K384 **(I)** and K388 **(J)**. The same statistical tests as in Fig. 2C were carried out; *, $p < 0.05$; **, $p < 0.01$; ***, $p < 0.001$; #, $p < 0.0001$. Data are presented as

Individual points and as mean ± SEM. The dotted lines represent the WT mean pH_{50} and pHD_{50} values.

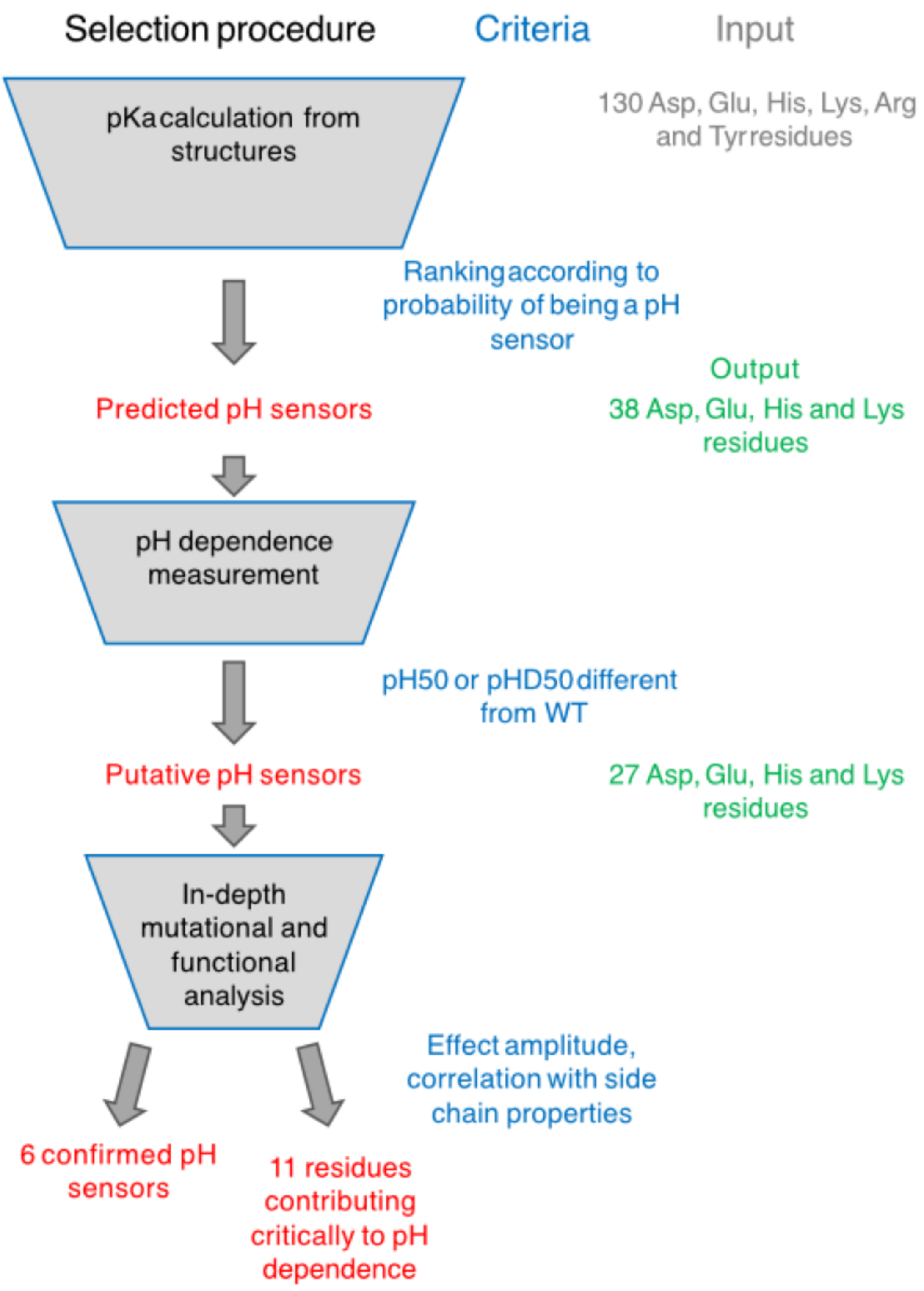

Supplementary Fig. 5. Schematic view of the workflow of computational and functional analyses of titratable residues. The scheme summarizes the workflow of the analyses and the selection criteria for different categories of residues contributing to ASIC pH dependence. Numbers of residues are indicated per subunit.

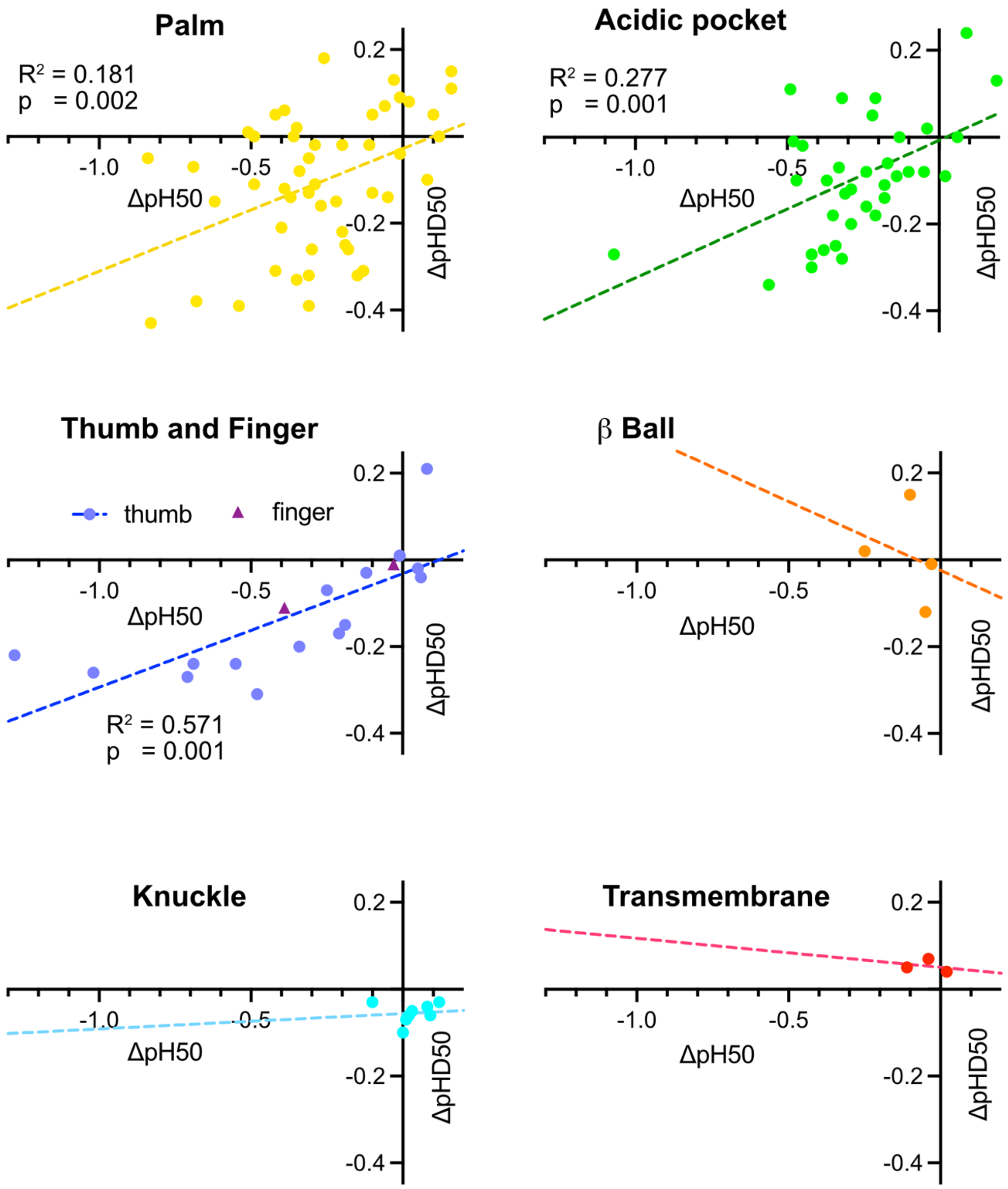

Supplementary Fig. 6. Correlation between mutation-induced shift in pH50 and pHD50. Each point in the figure panels represents one mutant, presented for the individual channel domains. The DpHD50 relative to the WT value is plotted as a function of the DpH50 relative to WT of the same mutant. The figure is based on the data of supplementary table 2.

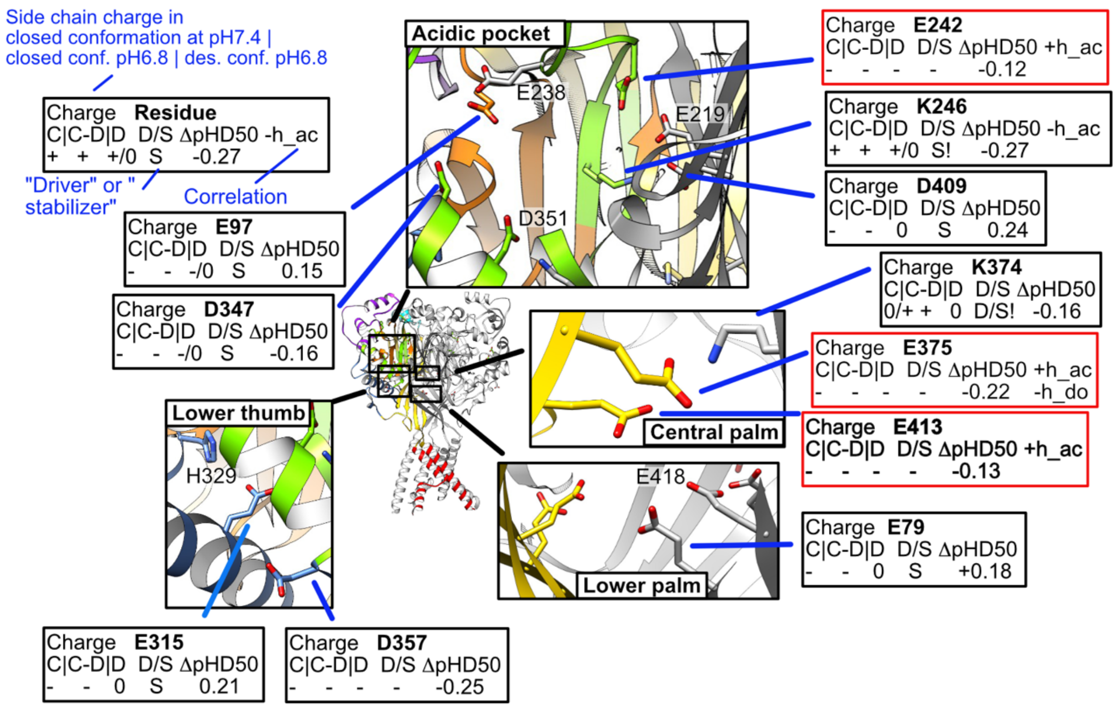

Supplementary Fig. 7. Hotspots of pH sensing for SSD. This figure summarizes the structural context, computational predictions and functional results of confirmed pH sensors and of putative pH sensors whose mutation strongly affected the pH dependence of SSD. The four H^+-sensing regions in the context of SSD, AcP, lower thumb, central palm and lower palm are shown in focused structural images, whose locations are indicated in an image of the entire ASIC1a structure (center). As illustrated in the upper left corner, black and red boxes provide the following information on the key residues: Charge, charge of the side chain based on the calculation of the protonation fraction (**Table 3**) for the situations C = closed structure, pH7.4; C-D, closed conformation, pH6.8 (initiation of transition); D, desensitized structure, pH6.8. D/S, based on the changes in protonation fraction, a residue is expected to drive the transition (D) or stabilize the desensitized state (S). An "!" indicates that lowering of the pH induces a deprotonation of this residue. ΔpHD_{50}, change in pHD_{50} of the conservative mutation to a non-titratable amino acid. Correlations to biophysical side chain properties (**Table 2**) are indicated with the + sign if the correlation is positive, - if there is a negative correlation; h_ac, hydrogen acceptor; h_do, hydrogen donor; coil, probability of occurring in coils. Red box, confirmed pH sensor; black box, putative pH sensor whose

conservative mutation to a non-titratable amino acid induces a strong effect on pH dependence.

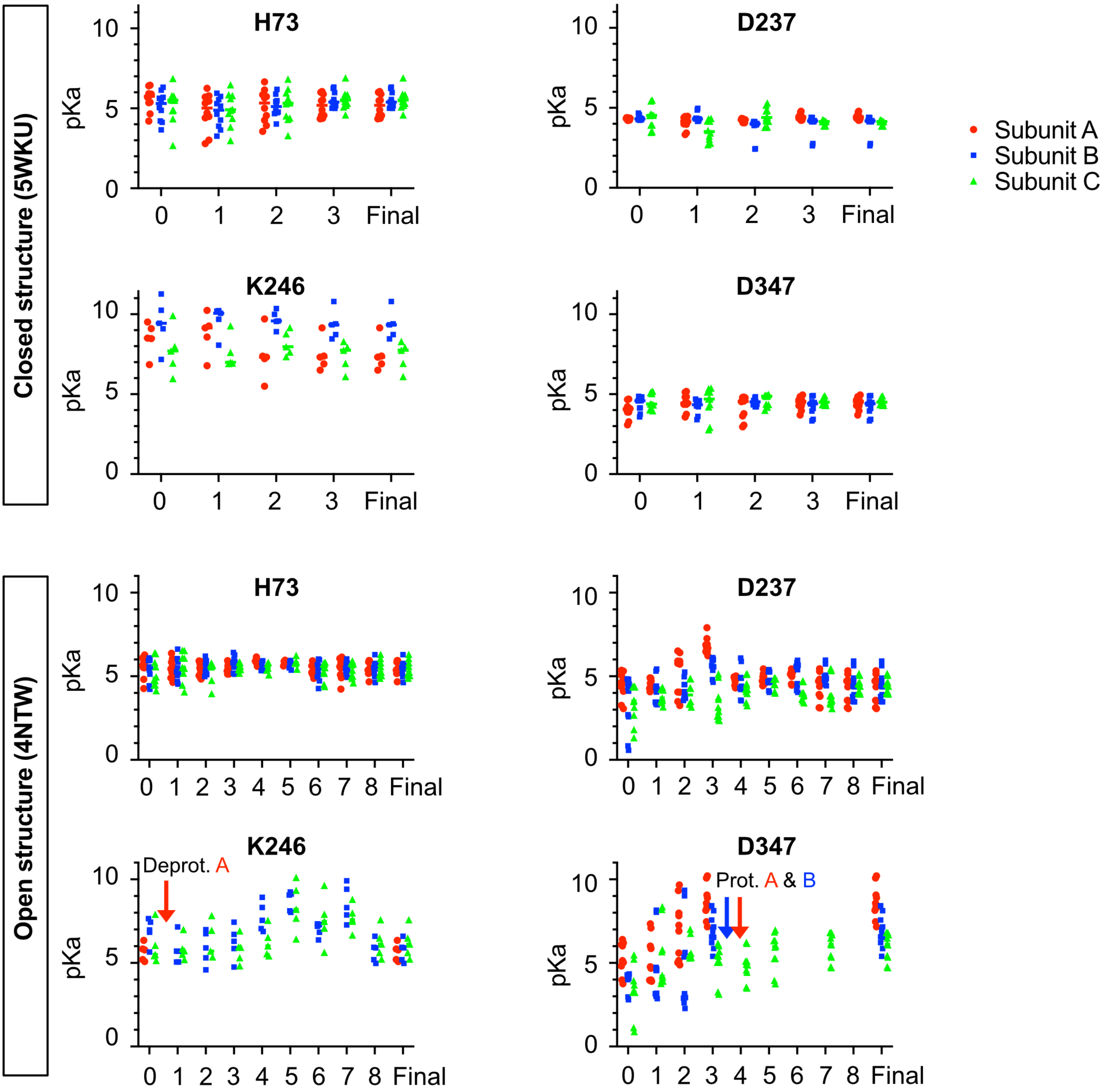

Supplementary Fig. 8. Evolution of pKa values as a function of protonation round for selected residues. pKa values calculated for residues H73, D237, K246 and D347 are shown for different rounds of calculation together with the finally calculated pKa value, for the three subunits. Between the rounds, protonation changes were introduced for some residues, shown by colored arrows, as detailed in the *Methods*. The upper panels show calculations from the closed state structural model, over 4 rounds, the lower panels show calculations from the open structural model, over 9 rounds.

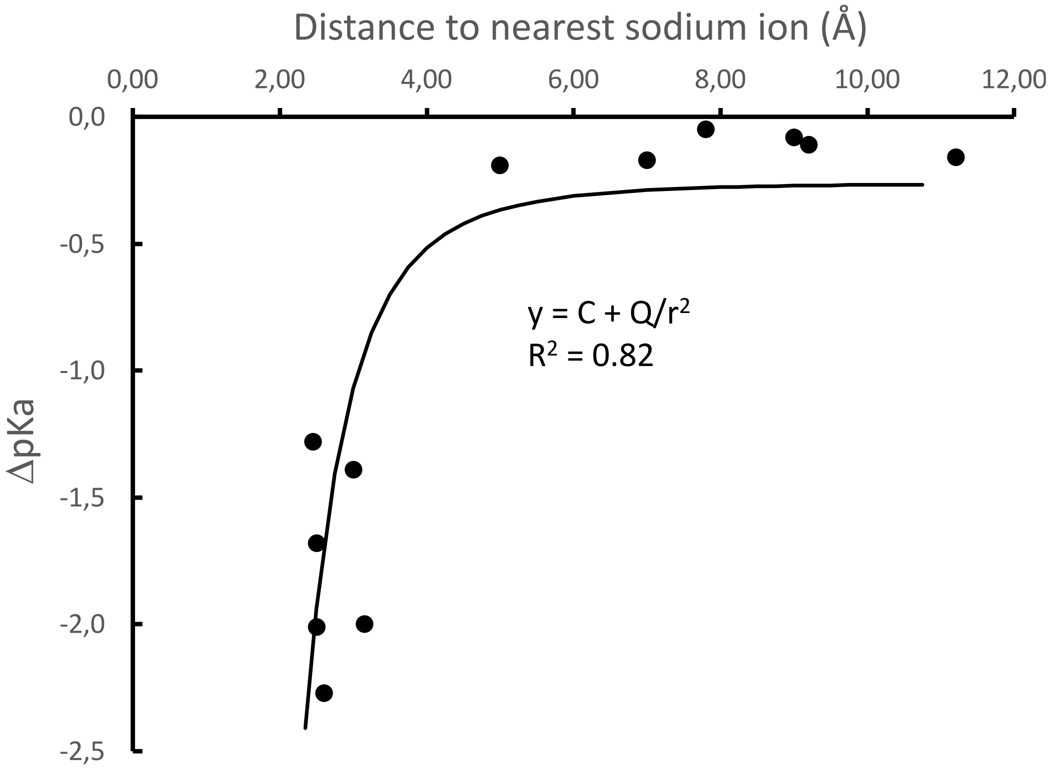

Supplementary Fig. 9. Distance dependence of monovalent cation effect on pKa calculation. The difference of the calculated pKa, with or without explicit consideration of a monovalent cation at the indicated distance, is shown, as detailed in the supplementary Methods section. Numerical values of the data are presented in supplementary table 4.

References

1 Bignucolo, O., Chipot, C., Kellenberger, S. & Roux, B. Galvani Offset Potential and Constant-pH Simulations of Membrane Proteins. *The journal of physical chemistry. B* **126**, 6868-6877 (2022). https://doi.org/10.1021/acs.jpcb.2c04593

2 Chatelain, F. C. *et al.* Mechanistic basis of the dynamic response of TWIK1 ionic selectivity to pH. *Nature communications* **15**, 3849 (2024). https://doi.org/10.1038/s41467-024-48067-w

3 Braun, N. *et al.* High-throughput characterization of photocrosslinker-bearing ion channel variants to map residues critical for function and pharmacology. *PLoS Biol* **19**, e3001321 (2021). https://doi.org/10.1371/journal.pbio.3001321

4 Paukert, M., Chen, X., Polleichtner, G., Schindelin, H. & Grunder, S. Candidate amino acids involved in H^+ gating of acid-sensing ion channel 1a. *J Biol Chem* **283**, 572-581 (2008). https://doi.org/10.1074/jbc.M706811200

5 Paukert, M., Babini, E., Pusch, M. & Grunder, S. Identification of the Ca^{2+} blocking site of acid-sensing ion channel (ASIC) 1: implications for channel gating. *J Gen Physiol* **124**, 383-394 (2004).

6 Liechti, L. A. *et al.* A combined computational and functional approach identifies new residues involved in pH-dependent gating of ASIC1a. *J Biol Chem* **285**, 16315-16329 (2010). https://doi.org/M109.092015 [pii] 10.1074/jbc.M109.092015

7 Bonifacio, G., Lelli, C. I. & Kellenberger, S. Protonation controls ASIC1a activity via coordinated movements in multiple domains. *J Gen Physiol* **143**, 105-118 (2014). https://doi.org/10.1085/jgp.201311053

8 Vullo, S., Ambrosio, N., Kucera, J. P., Bignucolo, O. & Kellenberger, S. Kinetic analysis of ASIC1a delineates conformational signaling from proton-sensing domains to the channel gate. *Elife* **10** (2021). https://doi.org/10.7554/eLife.66488

9 Bargeton, B. & Kellenberger, S. The contact region between three domains of the extracellular loop of ASIC1a is critical for channel function. *J Biol Chem* **285**, 13816-13826 (2010). https://doi.org/M109.086843 [pii] 10.1074/jbc.M109.086843

10 Krauson, A. J., Rued, A. C. & Carattino, M. D. Independent contribution of extracellular proton binding sites to ASIC1a activation. *J Biol Chem* **288**, 34375-34383 (2013). https://doi.org/10.1074/jbc.M113.504324

11 Molton, O., Bignucolo, O. & Kellenberger, S. Identification of the modulatory Ca(2+)-binding sites of acid-sensing ion channel 1a. *Open biology* **14**, 240028 (2024). https://doi.org/10.1098/rsob.240028
