# Supplementary material for: Molecular mechanisms and hotspots of pH sensing in ASIC1a revealed by computational and functional analysis
Source: Commun Biol. 2025 Nov 26;8:1692. doi: 10.1038/s42003-025-09090-9 (PMC12658276; doi:10.1038/s42003-025-09090-9)
Supplement: Supplementary file 3 — Description of Additional Supplementary Files [file 42003_2025_9090_MOESM3_ESM.pdf]

# Description of Additional Supplementary Files

File name- Supplementary Data 1

File description – Data underlying the correlation between side chain properties and mutant pH dependence that are presented in Table 2

File name- Supplementary Data 2

File description – Source data of Figures and tables
